# Managerial Competency and Perceived Organizational Support in Relation to Job Stress Among Nurse Managers in the Republic of Korea: A Cross-Sectional Study

**DOI:** 10.3390/healthcare14162526

**Published:** 2026-08-13

**Authors:** Eun Ju Lee, Se Young Jung

**Affiliations:** 1 Nursing Department, Dongnam Institute of Radiological & Medical Sciences, Busan 46033, Republic of Korea; ferresin11@naver.com; 2 Department of Nursing, Young-san University, Yangsan 50510, Republic of Korea

**Keywords:** nurse managers, managerial competency, perceived organizational support, job stress

## Abstract

**Background**: This study aimed to examine the levels of nursing managerial competency, perceived organizational support, and job stress among nurse managers, and to examine the factors associated with their job stress. **Methods:** The participants included 133 nurse managers from seven hospitals in B Metropolitan City. Data were collected using self-reported questionnaires including the Korean Occupational Stress Scale and scales measuring nursing managerial competency and perceived organizational support. Collected data were analyzed using descriptive statistics, *t*-tests or ANOVA, Pearson’s correlation, and hierarchical multiple regression, with participants’ general characteristics entered as covariates. **Results:** Mean scores were 4.16 (out of 5) for nursing managerial competency, 3.24 (out of 5) for perceived organizational support, and 2.31 (out of 4) for job stress. Job stress showed negative correlations with both nursing managerial competency (r = −0.57, *p* < 0.001) and perceived organizational support (r = −0.79, *p* < 0.001). After adjustment for general characteristics, hierarchical regression showed that both nursing managerial competency (β = −0.18, *p* = 0.009) and perceived organizational support (β = −0.70, *p* < 0.001) were significantly associated with job stress, and the model accounted for 65.6% of the variance. **Conclusions:** Nurse managers with higher managerial competency and organizational support perceptions reported lower job stress. Strategies to enhance nursing managerial competency and organizational support should be developed to effectively manage nurse managers’ job stress.

## 1. Introduction

### 1.1. Rationale

Nurse managers not only guide and manage clinical nurses to provide patients with high-quality care, but also play a central role in connecting front-line nurses with the hospital’s organizational structure [1,2]. The role is therefore inherently dual: nurse managers support and develop the nursing team while remaining accountable for the efficient delivery of unit-level services. However, modern management strategies for healthcare organizations prioritize efficiency, leading to increases in the work outcome indices demanded of nurse managers. This has led to an expansion of the management or administrative functions of nurse managers that, combined with pressure for achievement, increases job stress [3]. Persistent job stress among nurse managers can lead to health problems (e.g., dyspepsia, headache), decreased job satisfaction, and increased fatigue and turnover intention [3,4]. This ultimately reduces the efficiency of nursing organizations, increases staff nurse turnover rates, and reduces the quality of patient care. This can generate negative ripple effects throughout the organization, eventually leading to increased healthcare costs [5,6]. Hence, effectively mitigating job stress among nurse managers is crucial for the management of nursing organizations.

Factors associated with job stress among nurse managers can be broadly categorized as personal factors and organizational factors [7,8]. Personal factors include sex, work experience, education, and managerial competency, while organizational factors include hospital type, number of beds managed, role overload, understaffing, and lack of organizational support. According to previous studies, female nurse managers report higher job stress than male nurse managers, and nurse managers who have less managerial experience report higher job stress [8], with lower managerial competency being associated with increased job stress [2]. Other factors associated with increased job stress include working at a private hospital [9], a greater number of beds managed [7], a greater shortage in nursing staff [10], and greater role overload or role ambiguity [3]. Among these factors, managerial competency and perceived organizational support should be focused on as they can be improved through education and policy development.

The relationship between managerial tenure and job stress warrants particular attention. Evidence from several health systems indicates that stress is highest during the period of role transition that follows appointment, when newly appointed managers must assume administrative and personnel responsibilities for which their clinical training has not prepared them, and that it tends to decline as managerial experience accumulates and role clarity improves [8,11]. Other studies, however, report that stress does not decline monotonically and may increase again among long-tenured managers because of the cumulative burden of unrelieved demands [3,12]. This inconsistency indicates that managerial tenure cannot be assumed to operate uniformly across settings, and it provides part of the rationale for examining managerial experience alongside competency and organizational support in the present study.

Nursing managerial competency is the combined knowledge, attitude, skill, and behavior required in nurse management scenarios [13], and is associated with nurses’ job satisfaction, motivation, and quality of patient care [14]. In current healthcare environments, nurse managers are required to not only demonstrate clinical skills, but also to produce results in terms of management and administration, further highlighting the importance of managerial competency [1]. However, many nurse managers assume managerial roles without developing sufficient competency [8], which may leave them less able to cope with their various tasks and responsibilities and may therefore be accompanied by higher job stress.

Perceived organizational support refers to the belief among members of an organization regarding how much the organization values their contributions and is interested in their welfare [15]. Organizational support theory, based on social exchange theory, postulates that appropriate organizational support increases the work output of organization members, with favourable consequences for the organization [16]. Organizational support is associated with higher job motivation and satisfaction among nurses, lower job stress [17], and lower turnover rate and burnout, aiding the formation of a therapeutic environment [18]. However, for nurse managers exposed to excessive occupational loads and time pressure, a lack of organizational support is an important factor hindering effective role performance [19].

Previous studies on nurse managers in South Korea primarily focus on the association of nurse managers’ leadership with staff nurses [20] or with nurse managers’ work outcomes [14], and relatively few studies have explored job stress among nurse managers [12]. In particular, studies on perceived organizational support among nurse managers are few in number [21], and studies analyzing the relationship between nursing managerial competency and job stress are extremely limited. Moreover, there are almost no Korean studies simultaneously examining nursing managerial competency and perceived organizational support, making the development of practical intervention strategies for nurse managers challenging. Therefore, there is a need to systematically identify the factors associated with job stress among nurse managers, and to investigate their relationships, particularly focusing on nursing managerial competency and perceived organizational support, which are factors that can be improved at the organizational level.

Although the present study was conducted in South Korea, its relevance is not confined to that setting. Two considerations support this claim. First, the Korean context provides a useful example of a configuration of conditions that exists, in varying degrees, in many health systems. Nurse managers in Korea are typically appointed on the basis of clinical seniority, without formal managerial preparation or certification, and assume administrative accountability immediately upon appointment. The span of control is wide: participants in the present study were responsible for a mean of 38.9 beds and 23.2 nurses, which exceeds the figures reported in comparable studies conducted in Europe and North America [7,8]. Hospital accreditation requirements impose extensive documentation and performance-monitoring duties on unit managers, and organizational structures remain comparatively hierarchical, which may constrain the upward communication through which support is ordinarily sought. Studying nurse managers under these conditions therefore offers valuable insights into how personal and organizational resources relate to strain when demands are high and preparation is limited.

Second, the underlying phenomenon is not country-specific. Role overload, administrative burden, and elevated stress among first-line nurse managers have been documented across a wide range of health systems, including integrative review evidence spanning multiple countries [3] and cross-sectional studies conducted in Belgium and the United States [7,8,19]. What differs across settings is the magnitude of the demands and the availability of preparation and support, not the existence of the problem. Findings obtained where demands are high and preparation is limited are therefore likely to be informative for health systems facing nursing workforce shortages, widening spans of control, or increasing accreditation-related administrative requirements, and they may be of particular relevance to other health systems in which nurse managers are appointed from the clinical ranks without dedicated managerial training.

### 1.2. Purpose

The purpose of this study was to examine the levels of nursing managerial competency, perceived organizational support and job stress among nurse managers, and to examine the associations of nursing managerial competency and perceived organizational support with job stress. Both variables were selected because, unlike demographic or structural characteristics, they are amenable to change through education and organizational policy and therefore represent plausible targets for intervention. The findings are intended to provide baseline data for the development of strategies to mitigate job stress among nurse managers, thereby contributing to the stable management of nursing organizations and to the quality of patient care. The specific objectives were as follows:

(1) To describe the participants’ general characteristics.
(2) To measure the participants’ nursing managerial competency, perceived organizational support, and job stress.
(3) To compare differences in nursing managerial competency, perceived organizational support, and job stress based on general characteristics.
(4) To investigate correlations between nursing managerial competency, perceived organizational support, and job stress.
(5) To examine the associations of nursing managerial competency and perceived organizational support with job stress after adjustment for the participants’ general characteristics.

### 1.3. Conceptual Framework and Hypotheses

This study was guided by organizational support theory and the job demands–resources model. Organizational support theory, which is grounded in social exchange theory, proposes that employees form a global belief about the extent to which the organization values their contributions and cares about their well-being, and that this belief shapes their responses to workplace demands [15,16]. The job demands–resources model complements this perspective by distinguishing job demands, which deplete energy and give rise to strain, from resources, which buffer the effect of demands and support effective role performance [22]. Resources may be located within the individual, in the form of personal capabilities, or within the organization, in the form of support, recognition, and reward.

Within this framework, the nurse manager role is characterized by a combination of high demands—a wide span of control, extensive administrative responsibility, and simultaneous accountability to senior management and to frontline nurses—and two categories of resource that may attenuate the resulting strain. Nursing managerial competency, comprising the knowledge, attitudes, skills, and behaviours required to perform the managerial role [13], was conceptualized as a personal resource. Perceived organizational support, comprising the manager’s appraisal of the recognition and care extended by the institution [15], was conceptualized as an organizational resource. Job stress was treated as the indicator of strain. Participants’ general characteristics, which have been reported to covary with job stress in previous studies [7,8,12], were treated as covariates rather than as variables of primary interest.

Because the study employed a cross-sectional design, the hypotheses are stated as directional associations and are not intended to imply causal relationships. The hypotheses examined were as follows:

**H1.**  *Nursing managerial competency will be inversely associated with job stress among nurse managers*.

**H2.**  *Perceived organizational support will be inversely associated with job stress among nurse managers*.

**H3.**  *Nursing managerial competency and perceived organizational support will each show an association with job stress that is independent of the participants’ general characteristics*.

**Figure 1:**
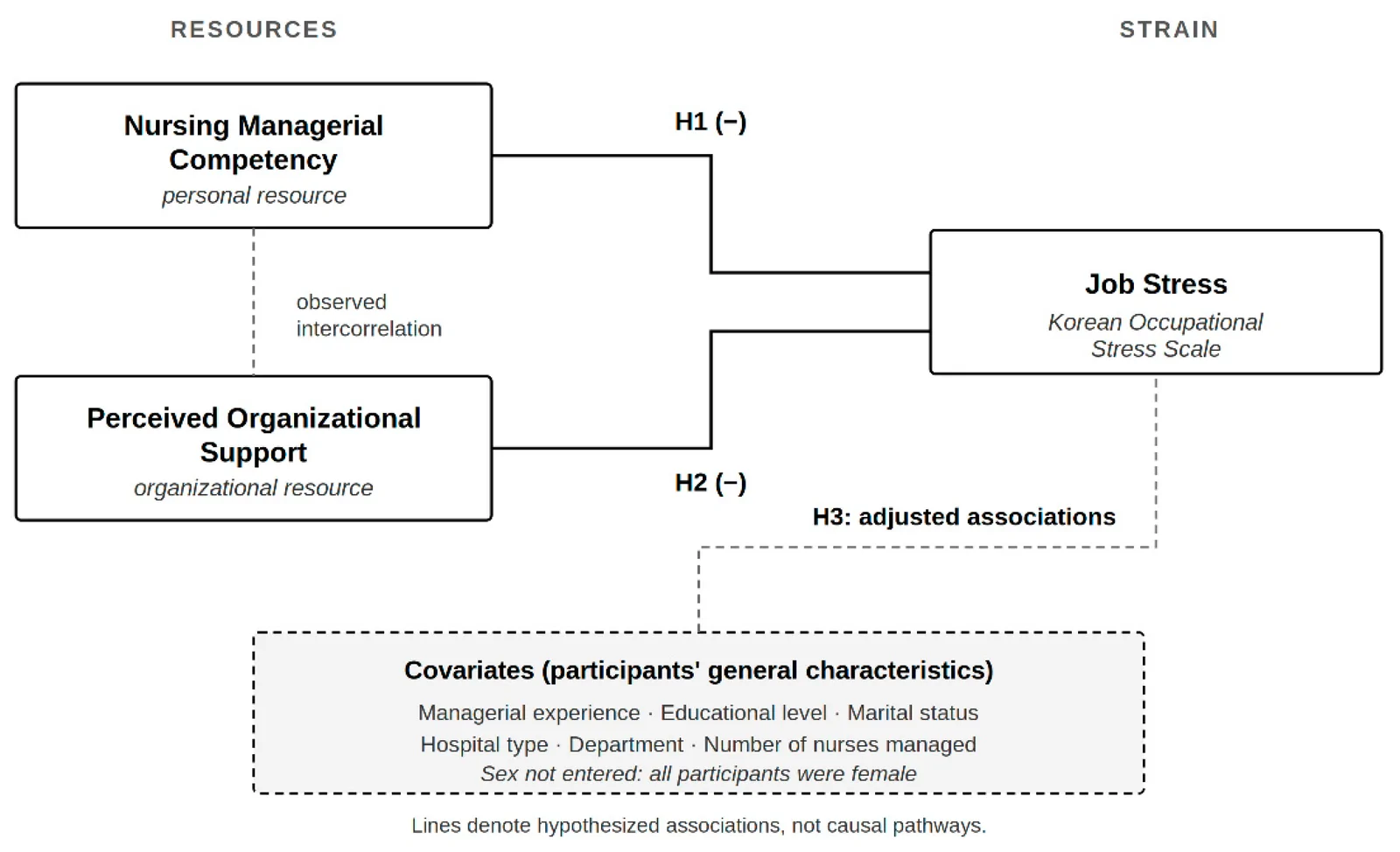
Conceptual framework of the study. Nursing managerial competency was conceptualized as a personal resource and perceived organizational support as an organizational resource, and both were hypothesized to be inversely associated with job stress (H1, H2). Participants’ general characteristics were entered as covariates, and each association was examined after adjustment for these characteristics (H3). Because the study used a cross-sectional design, the lines denote hypothesized associations and do not represent causal pathways.

The conceptual framework of the study is presented in Figure 1.

## 2. Materials and Methods

### 2.1. Study Design

This was a descriptive cross-sectional correlational study designed to examine the associations among nursing managerial competency, perceived organizational support and job stress among nurse managers at seven healthcare organizations in B City. Because the design is cross-sectional and all variables were measured concurrently, the study cannot establish the direction of the observed associations and no causal inference is intended.

### 2.2. Participants and Data Collection

#### 2.2.1. Participants and Inclusion Criteria

The participants in this study were head nurses (ward, ICU, ER) employed at one of three tertiary hospitals or four general hospitals (seven organizations in total) with 300 beds or more, located in B City. Nurse managers who understood the study objectives and procedure, and provided written consent to participate were included in the study. Participants were recruited by convenience sampling; no random or stratified selection was applied at either the institutional or the individual level. Hospital type was classified according to the designation held by each institution at the time of data collection.

#### 2.2.2. Sample Size Calculation and Participant Flow

The required sample size was determined by a priori power analysis using G*Power 3.1, which is a standard tool for computing the sample size required to detect a specified effect at a given level of power and is widely used for this purpose in nursing research. The analysis was specified for multiple linear regression (F-test, fixed model, R^2^ deviation from zero) with an effect size f^2^ = 0.15, corresponding to a medium effect by Cohen’s convention, α = 0.05, power = 0.80, and 10 predictors, yielding a minimum required sample of 118 participants. Anticipating an attrition rate of approximately 20% from ineligibility and incomplete responses, 147 questionnaires were distributed. The figure of 147 therefore refers to the number of questionnaires distributed rather than to a pre-existing population of that size.

Questionnaires were distributed only to nurse managers who had already provided written informed consent, and each completed questionnaire was returned to the researcher in a sealed envelope collected in person at the participant’s unit. All 147 distributed questionnaires were therefore returned. Of these, 8 were excluded because the respondent did not meet the inclusion criteria and 6 were excluded because of incomplete responses, leaving 133 questionnaires for the final analysis, corresponding to 90.5% of those distributed. The final sample exceeded the minimum of 118 required by the power analysis, and the number of predictors entered in the final regression model was consistent with the number assumed in the a priori calculation.

### 2.3. Instruments

This study used a self-report questionnaire comprising 86 items across the categories of general characteristics (8 items), nursing managerial competency (38 items), perceived organizational support (16 items), and job stress (24 items).

#### 2.3.1. General Characteristics

Eight items measured the participants’ sociodemographic and job-related characteristics: sex, age, education level, marital status, clinical experience, department, number of beds, and number of staff managed.

#### 2.3.2. Nursing Managerial Competency

An instrument based on the job description for ward nurse managers developed by Jo [13] and used by Kim [23] was employed to measure nursing managerial competency. This instrument consists of 38 items in total, divided into the domains of clinical nursing professional (11 items), leader (9 items), administrative manager (12 items), and educator (6 items). Each item is scored on a 5-point Likert scale, with higher scores indicating better nursing managerial competency. Cronbach’s α was 0.96 in the study by Kim [23], 0.96 in a study by Lee et al. [24], and 0.97 in our study.

#### 2.3.3. Perceived Organizational Support

Perceived organizational support was measured using a Korean version [25] of the Survey of Perceived Organizational Support developed by Eisenberger et al. [15]. The instrument authors gave permission to use the instrument. The version administered comprised 16 items. The original instrument uses a 7-point scale, but this was changed to a 5-point scale for our study, and six negative items were reverse-scored. Higher scores indicate a higher level of perceived organizational support. Cronbach’s α was 0.97 in the study by Eisenberger et al. [15], 0.89 for the Korean version of the scale [25], and 0.93 in our study.

#### 2.3.4. Job Stress

Job stress was measured using 24 items from the Korean Occupational Stress Scale (KOSS), developed by Chang et al. [26]. The scale is divided into 8 items measuring job-related factors (job demands, lack of job autonomy), 5 items measuring personal factors (job insecurity, relationship conflicts), and 11 items measuring organization-related factors (organizational structure, inadequate compensation, workplace culture). Each item is scored on a 4-point Likert scale (1 point = “completely disagree”–4 points = “strongly agree”), and some items are reverse scored. Higher total scores indicate a higher level of job stress. In accordance with the standard scoring procedure for the KOSS, each subscale score was converted to a 0–100 scale and the total score was computed as the mean of the converted subscale scores; this converted total was used as the dependent variable in the regression analysis. Cronbach’s α was 0.82 at the time of scale development and 0.83 in our study.

### 2.4. Data Collection

Data for this study was collected between 23 August and 30 October 2019. Following a telephone explanation of the study’s purpose and objectives to the healthcare organizations over telephone, permission for data collection was obtained in accordance with the internal research committee procedures at each hospital. We directly visited each hospital and distributed structured, self-report questionnaires. The questionnaire cover explained the study objectives and procedure, anonymity and privacy protection, and that participants could withdraw from the study at any time. Nurse managers who voluntarily agreed to participate completed the written consent form and subsequently completed the questionnaire, which took approximately 30–35 min. Participants were informed that they could contact a researcher via cell phone if they had any questions while responding to the questionnaire. After completing the questionnaire, the responses were collected in sealed envelopes to protect personal information. All respondents received a small gift as a token of gratitude.

### 2.5. Data Analysis

Collected data were analyzed using IBM SPSS Statistics 25.0 software (IBM Corp., Armonk, NY, USA) as follows:

Participants’ general characteristics, nursing managerial competency, perceived organizational support, and job stress were analyzed using frequencies, percentages, means, and standard deviations. Differences in variables based on general characteristics were analyzed using independent samples *t*-tests and ANOVA, and the Scheffé test was used for post hoc analysis. The associations among nursing managerial competency, perceived organizational support, and job stress were analyzed using Pearson correlation coefficients.

Hierarchical multiple regression analysis was conducted to examine the associations of nursing managerial competency and perceived organizational support with job stress after adjustment for the participants’ general characteristics. Job stress, expressed on the converted 0–100 scale, served as the dependent variable. Model 1 included the general characteristics presented in Table 1 as covariates: managerial experience (years, continuous), educational level (master’s degree or higher versus bachelor’s degree or lower), marital status (married versus single or other), hospital type (tertiary versus general), department (surgery and intensive care unit/emergency room, with internal medicine as the reference category), and number of nurses managed (continuous). Sex was not entered because all participants were female. Age, total clinical experience, and number of beds were not entered in the main model because they were highly collinear with the retained covariates, with variance inflation factors exceeding 5; a sensitivity analysis including these variables was conducted. Model 2 additionally included nursing managerial competency and perceived organizational support, and the increase in explained variance was evaluated using the F-change statistic. Regression assumptions were examined using the Durbin–Watson statistic, tolerance values, and variance inflation factors. Statistical significance was set at *p* < 0.05.

### 2.6. Ethical Considerations

The Institutional Review Board (IRB) at D Hospital in B Metropolitan City approved this study (D-1908-018-002). The participants were thoroughly informed of the study objectives and procedure, protection of personal information and guaranteed anonymity, voluntary participation and the right to withdraw, and that they would face no disadvantages if they refused to participate. Only participants who provided written consent were included in the final analysis. All collected data were used solely for research purposes and were securely stored and managed with password protection to ensure anonymity and confidentiality. Participants were provided with the contact details for the principal investigator for any questions or discomfort related to the study.

## 3. Results

### 3.1. General Characteristics of Participants

Overall, 133 participants were included in this study, all of whom were female nurse managers. The mean age was 46.55 ± 5.77 years, and 67.7% had a master’s degree or higher (Table 1). Most participants were married, and 54.9% were employed at a tertiary hospital. The mean total clinical experience was 24.48 ± 6.82 years, and the mean experience as a manager was 6.90 ± 5.99 years. The most common department was internal medicine, the mean number of beds per ward was 38.92 ± 13.36, and the mean number of nurses managed was 23.20 ± 10.78.

### 3.2. Nursing Managerial Competency, Perceived Organizational Support, and Job Stress

The mean score for nursing managerial competency was 4.16 ± 0.45 out of 5 points (Table 2). Among the subdomains, the administrative manager domain showed the highest score, followed by the leader domain, the clinical nursing professional domain, and the educator domain. Of the 38 total items, the competency that showed the highest score was ‘compiling work rotations and attendance management’ (4.51 ± 0.66 points), and the competency with the lowest score was ‘Educational materials/program development and instructor activities for nursing staff’ (3.83 ± 0.94 points).

The mean score for perceived organizational support was 3.24 ± 0.55 out of 5 points. By item, ‘The hospital acknowledges my contributions’ displayed the highest score (4.05 ± 0.80 points) and ‘I will be replaced if my salary can be reduced’ displayed the lowest score (2.92 ± 1.00 points, reverse scored).

The mean score for job stress was 2.31 ± 0.29 out of 4 points (converted to 42.25 ± 9.55 out of 100 points). Among the subdomains, ‘job-related factors’ displayed the highest score, followed by ‘organization-related factors’ and ‘personal factors’. By item, ‘I have to do several things at once’ displayed the highest score (3.24 ± 0.59 points).

### 3.3. Differences in Nursing Managerial Competency, Perceived Organizational Support, and Job Stress Based on General Characteristics

Nursing managerial competency showed significant differences based on age, clinical experience, and managerial experience (Table 3). In the post hoc tests (Scheffé), nursing managerial competency was significantly greater in the group aged 51–60 years than in the group aged 40 years or younger, significantly greater in the group with 31 years or more of clinical experience compared to the group with less than 20 years of experience, and significantly greater in the groups with 10 years or more of managerial experience or 3–10 years of managerial experience compared to the group with less than 3 years of experience.

Perceived organizational support showed significant differences based on the type of hospital and managerial experience. General hospital workers displayed higher perceived organizational support than tertiary hospital workers. In post hoc testing, the group with 10 years or more of managerial experience showed significantly higher perceived organizational support than the group with less than 3 years of experience. There were no significant differences in perceived organizational support based on age, education level, marital status, clinical experience, department, number of beds, number of nurses managed, or total number of staff managed.

Job stress showed significant differences based on managerial experience. In post hoc testing, the group with less than 3 years of managerial experience displayed significantly higher job stress than the group with 10 years or more of experience. There were no significant differences in job stress based on other general characteristics.

### 3.4. Correlations Between Nursing Managerial Competency, Perceived Organizational Support, and Job Stress

There was a significant positive correlation between nursing managerial competency and perceived organizational support (Table 4). Job stress showed significant negative correlations with both nursing managerial competency and perceived organizational support. In other words, higher perceived organizational support and higher nursing managerial competency led to reduced job stress.

### 3.5. Associations of Nursing Managerial Competency and Perceived Organizational Support with Job Stress

Hierarchical multiple regression analysis was conducted to examine factors associated with job stress (Table 5). The Durbin–Watson statistic was 2.04, indicating independence of the residuals, and tolerance values ranged from 0.62 to 0.94 with variance inflation factors ranging from 1.06 to 1.62, indicating no issues with multicollinearity.

Model 1, which included the participants’ general characteristics as covariates, was not statistically significant overall and accounted for 8.8% of the variance in job stress (F = 1.72, *p* = 0.111). Among the covariates, only managerial experience showed a significant inverse association with job stress (β = −0.25, *p* = 0.007).

In Model 2, nursing managerial competency and perceived organizational support were added to the covariates. Both variables showed significant inverse associations with job stress (nursing managerial competency: β = −0.18, *p* = 0.009; perceived organizational support: β = −0.70, *p* < 0.001), whereas managerial experience was no longer significantly associated with job stress (β = −0.01, *p* = 0.841). Model 2 accounted for 65.6% of the variance in job stress, an increase of 56.8 percentage points over Model 1, and this change was statistically significant (F change = 101.38, *p* < 0.001).

Perceived organizational support showed the largest standardized coefficient, followed by nursing managerial competency. These associations were independent of the participants’ general characteristics, and the estimates were essentially unchanged in a sensitivity analysis that additionally adjusted for age, total clinical experience, and number of beds. Hypotheses H1, H2, and H3 were therefore supported.

## 4. Discussion

The purpose of this study was to examine the levels of nursing managerial competency, perceived organizational support and job stress among nurse managers, and to examine the associations of nursing managerial competency and perceived organizational support with job stress, thereby providing basic data for the management of job stress among nurse managers.

The mean nursing managerial competency score in this study was higher than the score in a study by Lee which used the same instrument [24]. The results aligned with a study by Kang et al. [2], in which nurse managers at tertiary hospitals reported higher competency overall due to educational programs to strengthen competencies and more opportunities to participate in research and projects. Among the subdomains, the administrative manager domain displayed the highest scores, followed by the leader, clinical professional, and educator domains. This aligns with studies by Kim [23] and Lee et al. [24] that used the same instrument, in which the administrative manager domain showed the highest scores. These results support the assertion that administrative tasks account for a gradually increasing portion of nurse managers’ workload in clinical settings, and that competency in this area is relevant for practice. However, the relatively low scores in the educator domain suggest that nurse managers do not fulfill the requirements for the development of educational materials and management of programs for nursing staff, or participation in external education. Hence, there is a need to develop customized programs to improve educator competencies among nurse managers, and to increase opportunities for participation in this area. Moreover, in South Korea, there is still a shortage of research that develops instruments for measuring nursing managerial competency tailored to hospital nurse managers [27]. Therefore, there is a need to develop additional instruments to measure nursing managerial competency that reflect diverse clinical settings.

Nursing managerial competency showed significant differences based on age and managerial experience. Nurse managers aged 51–60 years showed significantly higher managerial competency than those aged 40 years or younger or those aged 41–50 years. In addition, nurse managers with at least 10 years of managerial experience showed significantly higher competency than those with less than 3 years of experience or those with 3–10 years of experience. These findings are consistent with previous studies, which show that the accumulation of age and managerial experience was associated with a gradual improvement in nursing managerial competency via diverse managerial experiences [23,24]. Therefore, it is important to introduce systematic educational programs for improving competency among nurses who have been newly appointed as managers, and to explore strategies to support the long-term development of competency through collaboration with specialist educational organizations.

The mean perceived organizational support score was higher than the score reported by staff nurses [21], but lower than the score reported by nurse managers in the US [28]. This can be considered to align with the results of a study by Choi et al. [29], in which a higher position within an organization was associated with a trend for higher perceived organizational support. This suggests that the construction of fair treatment and compensation systems within organizations and improvements in working environments are important factors for improving perceived organizational support among nurse managers. In particular, ‘The organization would hire another person at a lower salary if they could’ received a low score, which indirectly suggests that some nurse managers have negative perceptions of the workforce management policies at their hospital. This indicates the need for reviews of staffing and staff compensation systems at the organizational level.

In our study, the mean job stress score for nurse managers was similar to the score reported in a study by Lee et al. [24]. Among subdomains, the ‘job demands’ domain showed the highest score, and items such as ‘I have to do several things at once’ and ‘I’m under time pressure because there is a high workload’ also showed high scores, which is consistent with reports that an excessive workload and time pressure are major correlates of stress among nurse managers [19,30]. Excessive work burdens have been associated with decreased job satisfaction and with a decline in the quality of patient care. Therefore, multifaceted efforts are required at the organizational level to maintain an appropriate workload, such as staff reallocation, clarification of roles, and the construction of efficient time management systems.

When hierarchical regression analysis was performed, managerial experience showed a significant association with job stress in the first model. However, when nursing managerial competency and perceived organizational support were included in the second model, managerial experience was no longer significantly associated with job stress and the total explanatory power increased to 65.6%. This is consistent with previous studies reporting that lower nursing managerial competency is accompanied by greater difficulty in alleviating work-related burdens [11], and that lower perceived organizational support is associated with increased job stress due to the loss of work motivation and insufficient psychological support [17,31]. Importantly, these associations persisted after adjustment for the participants’ general characteristics, and the covariate-only model was not statistically significant, which indicates that the observed associations are not attributable to the measured demographic or structural characteristics of the sample. As first-line managers for nursing organizations within hospitals, nurse managers act as a key link between the organization, nurses, and patients. Therefore, it is important to integrate systematic education for developing competency into practice, to construct fair compensation systems, and to form a positive organizational culture that may help to limit job stress among nurse managers.

### 4.1. The Korean Nurse Manager Context

The pattern of findings observed here is best understood in relation to the specific structural conditions under which nurse managers work in Korea. Participants in this study were responsible for a mean of 38.9 beds and 23.2 nurses. Spans of control of this magnitude exceed those reported in cross-sectional studies of first-line nurse managers conducted in Belgium and the United States [7,8], and they mean that the personnel, scheduling, and performance-monitoring responsibilities of a Korean unit manager are distributed across a substantially larger unit. It is therefore unsurprising that the ‘job demands’ domain of the KOSS produced the highest subscale score, and that the two highest-scoring items concerned simultaneous task demands and time pressure.

The internal profile of managerial competency observed in this study is consistent with the same structural account. The administrative manager domain received the highest scores and the educator domain the lowest. Rather than indicating a general strength in administration, this pattern plausibly reflects where managerial effort is directed: hospital accreditation in Korea imposes extensive documentation, indicator-monitoring, and reporting duties that fall largely on unit managers, and these duties are recurrent and externally audited. Educational activities for staff, by contrast, are neither externally audited nor protected within the working day, and are therefore the first to be displaced when administrative demand rises. The competency profile of Korean nurse managers may thus be shaped less by individual preference than by what the accreditation environment requires them to prioritise.

This structural reading also helps to explain the dual role that characterises the Korean nurse manager. Unit managers in Korea retain a substantial clinical presence while simultaneously carrying administrative accountability, and they are appointed on the basis of clinical seniority rather than managerial preparation. Managerial competency in this setting is therefore acquired largely through experience after appointment, which is consistent with our finding that competency increased with age and managerial tenure while job stress was highest among those with less than three years in the role. The transition into the managerial role, rather than the role itself, appears to be the period of greatest vulnerability.

Finally, perceived organizational support in our sample was lower than that reported among nurse managers in the United States [28], although higher than that reported among Korean staff nurses [21]. Two features of the Korean hospital environment may bear on this finding. Organizational structures remain comparatively hierarchical, which may limit the upward communication through which recognition and support are ordinarily sought, and reward systems for nurse managers are frequently tied to institutional performance indicators rather than to the managerial work itself. The low score obtained by the item concerning replacement at a lower salary is consistent with this interpretation.

Not all of these features are unique to Korea. Wide spans of control, accreditation-driven administrative burden, and appointment without managerial preparation are reported in many health systems, and the integrative review evidence indicates that first-line nurse manager stress is an international phenomenon [3]. What the Korean setting offers is a context in which these conditions occur together and with unusual intensity, which makes it a useful case for examining how personal and organizational resources relate to strain. The practical implication—that preparation for the managerial role and visible organizational support are the most modifiable points of intervention—is likely to apply wherever these conditions obtain.

### 4.2. Preparation for the Managerial Role

Two findings converge on the question of how nurse managers are prepared for their role. The educator domain received the lowest competency scores of the four domains, and nurse managers with less than three years of managerial experience reported both the lowest competency and the highest job stress. Taken together, these findings suggest that the period immediately following appointment is one in which managerial demands are encountered without a corresponding foundation of managerial preparation.

At present, there is no nationally standardised preparatory curriculum for newly appointed nurse managers in Korea, and preparation is largely institution-dependent and delivered after the manager has already assumed the post. This contrasts with settings in which structured competency frameworks define the knowledge and skills expected of the nurse manager role and provide a basis for both preparatory education and ongoing assessment. Evidence from role-transition research indicates that structured onboarding and mentorship for newly appointed nurse managers is associated with more favourable role transition [11]. The development of a preparatory curriculum organised around defined managerial competencies—including the educator competencies that scored lowest in this study—therefore represents a plausible and testable target for intervention, and one that could be evaluated in future prospective work.

### 4.3. Limitations

This study had several limitations. First, because the participants were limited to nurse managers from seven healthcare organizations in a specific region, the generalizability of the results may be limited. Second, the KOSS, which was used to measure job stress, was developed for use in general workers. Hence, it may not thoroughly reflect the unique characteristics of hospital work environments. Third, the use of a cross-sectional design limits the ability to establish causal relationships.

Fourth, unmeasured confounding cannot be excluded. Although the regression models were adjusted for the participants’ general characteristics, variables that were not measured in this study—including nurse staffing levels, organizational culture, the leadership style of senior nursing management, personality characteristics, and work–family conflict—may be associated with both the study variables and job stress. Residual confounding may therefore remain despite adjustment.

Fifth, participants were recruited by convenience sampling rather than by random or systematic selection, and all were drawn from institutions with 300 or more beds within a single metropolitan area. Nurse managers who chose to participate may differ systematically from those who did not, and the sample may not represent nurse managers working in smaller institutions or in other regions. Selection bias must therefore be considered when interpreting the findings.

Sixth, the direction of the observed associations cannot be determined, and reverse associations are plausible. Nurse managers experiencing high job stress may appraise organizational support less favourably, and may also evaluate their own managerial competency more critically, rather than low support and low competency being antecedent to stress. Longitudinal designs are required to distinguish between these possibilities.

Seventh, all variables were measured by self-report from the same respondents at a single time point, which raises the possibility of common method bias and may have inflated the observed associations. Harman’s single-factor test was conducted post hoc by entering all items into an unrotated principal component analysis; the first component accounted for 31.6% of the total variance, which is below the conventional threshold of 50%. This suggests that common method bias was unlikely to be a serious threat, but it cannot be entirely ruled out, and future studies would benefit from the inclusion of objective or informant-reported indicators.

Finally, the data were collected in 2019 and therefore precede the substantial changes in hospital work environments associated with the COVID-19 pandemic. The levels reported here should be interpreted with this in mind, although the associations among the study variables are less likely to have been affected.

Therefore, future studies should include nurse managers from different hospital sizes or from hospitals in diverse regions, and develop and implement job stress tools that account for the unique nature of healthcare organizations to design appropriate intervention strategies.

## 5. Conclusions

In this study, nursing managerial competency was higher among nurse managers of greater age and longer managerial experience. Perceived organizational support and nursing managerial competency were both significantly and inversely associated with job stress, and these associations were independent of the participants’ general characteristics. The findings suggest that nurse managers who perceive less organizational support and who report lower managerial competency may also report higher levels of job stress; however, because the design was cross-sectional, the direction of these associations cannot be determined.

Based on the results of this study, the following proposals can be made. First, a specialized scale should be developed that can measure nursing managerial competency and job stress in greater detail among nurse managers employed in hospitals in South Korea. Second, as discussed above, the absence of structured preparation for newly appointed nurse managers, together with the low scores obtained in the educator domain, indicates a need for a preparatory curriculum organised around defined managerial competencies, together with opportunities for continuing external education. Third, to enhance perceived organizational support among nurse managers, organizations should consider systematic strategies, including fair assessment and compensation systems, welfare promotion, and improvements to work environments. In addition, as the data for this study was collected using convenience sampling of general hospitals in a given region, it is important to conduct replication studies with more nurse managers to improve the generalizability of the results.

In conclusion, this study indicates that nursing managerial competency and perceived organizational support are associated with job stress among nurse managers. These findings may inform the development of strategies for reducing job stress in nurse managers, although their effectiveness would need to be evaluated in longitudinal or interventional research.

## Figures and Tables

**Table 1:** General Characteristics (N = 133).

Characteristic	Category	n	%	Mean ± SD
Sex	Male	-	-	
	Female	133	100.0	
Age(yr)	≤40	23	17.3	46.55 ± 5.77
	41–50	65	48.9	
	51–60	45	33.8	
Education level	Bachelor’s or lower	43	32.3	
	Master’s or higher	90	67.7	
Marital status	Married	116	87.2	
	Single or others	17	12.8	
Type of hospital	General hospital	60	45.1	
	Tertiary hospital	73	54.9	
Total clinical experience (yr)	≤20	46	34.6	24.48 ± 6.82
	20–30	46	34.6	
	≥30	41	30.8	
Management experience (yr)	<3	39	29.3	6.90 ± 5.99
	3–<10	60	45.1	
	≥10	34	25.6	
Department	Internal medicine	60	45.1	
	Surgery	47	35.3	
	ICU/ER	26	19.5	
Number of beds	≤30	29	21.8	38.92 ± 13.36
	31–50	84	63.2	
	≥50	20	15.0	
Number of nurses managed	≤20	60	45.1	23.20 ± 10.78
	≥21	73	54.9	
Number of staff managed	≤25	81	60.9	26.27 ± 12.91
	≥26	52	39.1	
Total		133	100.0	

**Table 2:** Descriptive Statistics of the Study Variables (N = 133).

Variables	Range	Items (n)	M ± SD
Nursing managerial competency	1~5	38	4.16 ± 0.45
Clinical Expert	1~5	11	4.14 ± 0.43
Leadership	1~5	9	4.14 ± 0.48
Administrative	1~5	12	4.26 ± 0.48
Educator	1~5	6	4.01 ± 0.63
Perceived organizational support	1~5	8	3.24 ± 0.55
Job stress	1~4	24	2.31 ± 0.29
Job demand	1~4	8	2.51 ± 0.21
Personal Factors	1~4	5	1.93 ± 0.40
Organizational Factors	1~4	11	2.34 ± 0.37

**Table 3:** Differences in Nursing Managerial Competency, Perceived Organizational Support, and Job Stress According to General Characteristics (N = 133).

Characteristic	Category	n	Nursing Managerial Competency			Perceived Organizational Support			Job Stress		
			Mean ± SD	t/F	*p* (Scheffe)	Mean ± SD	t/F	*p* (Scheffe)	Mean ± SD	t/F	*p* (Scheffe)
Age	≤40 ^a^	23	4.04 ± 0.44	7.36	0.001	3.11 ± 0.55	2.70	0.071	2.31 ± 0.33	2.06	0.131
	41–50 ^b^	65	4.06 ± 0.44		c > a,b	3.19 ± 0.60			2.30 ± 0.31		
	51–60 ^c^	45	4.36 ± 0.40			3.39 ± 0.43			2.20 ± 0.20		
Education level	Bachelor’s or lower	43	4.15 ± 0.49	−0.16	0.877	3.22 ± 0.62	−0.37	0.712	2.29 ± 0.32	0.62	0.533
	Master’s or higher	90	4.16 ± 0.43			3.26 ± 0.51			2.26 ± 0.27		
Marital status	Married	116	4.16 ± 0.43	0.38	0.703	3.25 ± 0.50	0.01	0.930	2.26 ± 0.27	−0.36	0.722
	Single or others	17	4.12 ± 0.56			3.23 ± 0.80			2.29 ± 0.39		
Type of hospital	General hospital	60	4.19 ± 0.45	0.74	0.460	3.38 ± 0.52	2.71	0.008	2.26 ± 0.25	−1.62	0.108
	Tertiary hospital	73	4.13 ± 0.45			3.13 ± 0.54			2.35 ± 0.31		
Total clinical experience (yr)	≤20 ^a^	46	4.02 ± 0.50	4.12	0.008	3.11 ± 0.62	1.90	0.133	2.33 ± 0.35	1.70	0.188
	20–30	46	4.14 ± 0.35		c > a	3.28 ± 0.51			2.25 ± 0.25		
	≥30 ^c^	41	4.34 ± 0.43			3.36 ± 0.48			2.22 ± 0.24		
Management experience(yr)	<3 ^a^	39	3.94 ± 0.55	10.92	<0.001	3.08 ± 0.68	3.12	0.047	2.36 ± 0.36	3.43	0.035
	3–<10 ^b^	60	4.16 ± 0.32		c > a,b	3.26 ± 0.48		c > a	2.26 ± 0.26		a > c
	≥10 ^c^	34	4.40 ± 0.40			3.40 ± 0.43			2.18 ± 0.22		
Department	Internal medicine	60	4.17 ± 0.45	0.10	0.904	3.27 ± 0.52	0.19	0.829	2.27 ± 0.27	0.57	0.570
	Surgery	47	4.13 ± 0.51			3.21 ± 0.65			2.29 ± 0.34		
	ICU/ER	26	4.17 ± 0.33			3.24 ± 0.39			2.22 ± 0.22		
Number of beds	≤30	29	4.26 ± 0.42	1.03	0.361	3.30 ± 0.43	0.41	0.664	2.21 ± 0.23	1.09	0.338
	31–50	84	4.14 ± 0.47			3.21 ± 0.59			2.29 ± 0.31		
	≥50	20	4.10 ± 0.39			3.30 ± 0.53			2.25 ± 0.27		
Number of nurses managed	≤20	60	4.17 ± 0.54	0.37	0.710	3.28 ± 0.61	0.68	0.495	2.26 ± 0.32	−0.32	0.749
	≥21	73	4.14 ± 0.36			3.21 ± 0.48			2.27 ± 0.26		
Number of staff managed	≤25	82	4.19 ± 0.48	0.92	0.330	3.22 ± 0.59	−0.37	0.709	2.27 ± 0.30	0.39	0.723
	≥26	51	4.11 ± 0.39			3.27 ± 0.48			2.26 ± 0.26		

**Table 4:** Correlation Matrix of Nursing Managerial Competency, Perceived Organizational Support, and Job Stress (N = 133).

Concept	Nursing Managerial Competence	Perceived Organizational Support
Nursing managerial competence	1	
Perceived organizational support	0.546	1
	(<0.001)	
Job stress	−0.566	−0.787
	(<0.001)	(<0.001)

**Table 5:** Hierarchical Multiple Regression Analysis Predicting Job Stress (N = 133).

Predictor	Model 1					Model 2				
	B	SE	β	t	*p*	B	SE	β	t	*p*
(constant)	45.15	3.74		12.07	<0.001	99.31	5.46		18.20	<0.001
Managerial experience(yr)	−0.39	0.14	−0.25	−2.75	0.007	−0.02	0.10	−0.01	−0.20	0.841
Education (≥master’s)	−1.81	1.80	−0.09	−1.00	0.317	−0.49	1.12	−0.02	−0.44	0.660
Marital status (married)	−0.30	2.50	−0.01	−0.12	0.905	−0.76	1.55	−0.03	−0.49	0.625
Hospital type (tertiary)	1.48	1.71	0.08	0.87	0.388	−0.97	1.09	−0.05	−0.89	0.375
Department (surgery)	0.05	1.85	0.00	0.03	0.977	−0.13	1.15	−0.01	−0.11	0.912
Department (ICU/ER)	−3.18	2.47	−0.13	−1.29	0.200	−2.35	1.53	−0.10	−1.53	0.129
Number of nurses managed	0.05	0.09	0.05	0.53	0.596	0.03	0.05	0.03	0.47	0.638
Nursing managerial competency						−3.84	1.44	−0.18	−2.67	0.009
Perceived organizational support						−12.19	1.14	−0.70	−10.66	<0.001
Adj-R^2^	0.037					0.630				
R^2^	0.088					0.656				
ΔR^2^	-					0.568				
F(*p*)	1.72(0.111)					26.01 (<0.001)				
F-changed (*p*)	-					101.38 (<0.001)				

Note. Job stress was expressed on a 0–100 scale, with higher scores indicating greater job stress. Reference categories were bachelor’s degree or lower for education, single or other for marital status, general hospital for hospital type, and internal medicine for department. Sex was not entered because all participants were female. Age, total clinical experience, and number of beds were not entered in the main model because of high collinearity with the retained covariates (variance inflation factors > 5); a sensitivity analysis including these variables yielded comparable estimates (nursing managerial competency β = −0.19, *p* = 0.007; perceived organizational support β = −0.69, *p* < 0.001). Durbin–Watson = 2.04; tolerance = 0.62–0.94; variance inflation factors = 1.06–1.62. SE = standard error; β = standardized coefficient.

## Data Availability

The original contributions presented in this study are included in the article. Further inquiries can be directed to the corresponding author.
